# Loss of Innervation and Axon Plasticity Accompanies Impaired Diabetic Wound Healing

**DOI:** 10.1371/journal.pone.0075877

**Published:** 2013-09-30

**Authors:** Chu Cheng, Vandana Singh, Anand Krishnan, Michelle Kan, Jose A. Martinez, Douglas W. Zochodne

**Affiliations:** Department of Clinical Neurosciences and the Hotchkiss Brain Institute, University of Calgary, Calgary, Alberta, Canada; Boston Children’s Hospital and Harvard Medical School, United States of America

## Abstract

Loss of cutaneous innervation from sensory neuropathy is included among mechanisms for impaired healing of diabetic skin wounds. The relationships between cutaneous axons and their local microenvironment during wound healing are challenged in diabetes. Here, we show that secondary wound closure of the hairy dorsal skin of mice is delayed by diabetes and is associated with not only a pre-existing loss of cutaneous axons but substantial retraction of axons around the wound. At 7d following a 3mm punch wound, a critical period of healing and reinnervation, both intact skin nearby the wound and skin directly at the wound margins had over 30-50% fewer axons and a larger deficit of ingrowing axons in diabetics. These findings contrasted with a pre-existing 10-15% deficit in axons. Moreover, new diabetic ingrowing axons had less evidence of plasticity. Unexpectedly, hair follicles adjacent to the wounds had a 70% reduction in their innervation associated with depleted expression of hair follicular stem cell markers. These impairments were associated with the local upregulation of two established axon regenerative ‘roadblocks’: PTEN and RHOA, potential but thus far unexplored mediators of these changes. The overall findings identify striking and unexpected superimposed cutaneous axon loss or retraction beyond that expected of diabetic neuropathy alone, associated with experimental diabetic skin wounding, a finding that prompts new considerations in diabetic wounds.

## Introduction

In human diabetes, ulcer wounds with impaired healing contribute to the risk of lower limb amputation [1]. The cumulative lifetime incidence of foot ulceration in diabetic subjects may reach 25%, identifying it as a very large health care burden [2]. Risk factors are neuropathy, microvascular disease, and macrovascular disease. Neuropathy is associated with heightened risk of damage to insensitive, injury-prone and anhidrotic feet [3,4].

It is uncertain whether alterations in skin and subdermal innervation alter the capability of new wounds to heal and how this innervation fares at injury sites during attempted repair. Wound healing depends on a series of reparative events, a number of which are known to be impaired in diabetes. For example, diabetes targets multiple growth factor, inflammatory, angiogenic and extracellular membrane proteins and pathways, each of which may have major impacts on the sequential stages of wound repair: fibrin clot formation, early and late inflammation, re-epithelialization, angiogenesis, granulation tissue formation, wound contraction, and scar formation [5,6]. For example, bidirectional signaling between the nervous and immune system may be critical in wound repair, as emphasized by Pradhan, Veves and colleagues [7]. Advanced glycosylation endproducts (AGEs), deposited widely in diabetic tissues and acting on RAGE (receptor for AGE) also alter several facets of the wound healing response including the sensory innervation of the skin [8]. Previous work has identified slow healing of experimental diabetic skin lesions [3].

Intact innervation facilitates wound healing, at least in part through the local release of active neuropeptides such as Substance P (SP) and calcitonin gene-related peptide (CGRP). SP and CGRP influence several facets of wound repair including local microvascular responses, signaling actions on inflammatory cells, fibroblast function, angiogenesisis, and perhaps wound innervation in conjunction with growth factors [9]. The neuropeptide content of healing diabetic wounds is attenuated, a feature that contributes to wound repair [10–13].

In this work, we analyzed axonal plasticity of cutaneous wounds in the dorsal hairy skin of mice with or without experimental diabetes. We identified a striking loss of axons, well beyond baseline axon loss, in the margins of the wound, a deficit of newly ingrowing axons and loss of perifollicular axons. An important mechanistic association with rises in wound RHOA and PTEN, known to inhibit nerve and wound plasticity, was identified [14,15].

## Materials and Methods

### (i) Mice, Diabetes biopsy

The mice used were CD-1 mice (Charles River, Wilmington, MA) or Swiss Webster (CFW, Charles River, Wilmington, MA), as characterized in our laboratory [16–18]. The protocol was reviewed and approved by the Animal Care Committee, University of Calgary. At 6 weeks of age, mice were injected with streptozotocin (STZ) over 3 days (85, 70 and 55 mg/kg; dissolved in citrate buffer) or citrate buffer alone. Diabetes (defined as fasting glucose ≥ 16mmol/L) was confirmed one and two months later. At two months of diabetes, mice were shaved and dorsal skin was collected with a 3mm disposable biopsy punch (ACU Punch; Acuderm Inc., Fort Lauderdale, FLA) under pentobarbital anaesthesia (65 mg/kg ip) _without the use of local anaesthetic. The biopsy sites were regularly cleansed with poloxamer-iodine complex 1% (West Penetone Inc, Montreal, Canada) but no antibiotics were administered. Seven days after the first biopsy, samples were analyzed at the site of the previous wound, its margins and in nearby normal skin.

### (ii) Immunohistochemistry and imaging analysis

Skin samples were fixed in 2% PLP (paraformaldehyde, lysine and periodate) for 16-20h at 4°C and cryoprotected overnight in 20% glycerol/0.1M Sorrenson phosphate buffer at 4°C as published [19,20]. Skin sections were of 25 μM thickness and washed in PBS, 1% triton X, incubated in blocking solution (10% goat serum,1% BSA, 0.05% NaN3, 0.3% TritonX100, 0.05% Tween20in PBS) for 1 hr at room temperature. Primary antibodies applied overnight at 4°C: PGP9.5 (rabbit polyclonal; 1:1000, Encor [MCA-BHT]); GAP43/B50 (rabbit polyclonal; 1:500,Abcam [#: ab11136]); PTEN (rabbit polyclonal; 1:50 Santa Cruz [SC-6817]), Lgr6 (rabbit polyclonal; 1:50, Santa Cruz [SC-99123]) as previously validated [14,17,21]. Secondary antibodies were applied and incubated for 1h at room temperature: Cy3 (Goat & Rabbit; Cedarlane) 1:100; AlexaFluor 488 (Goat; Invitrogen) 1:200. Sections were washed in PBS and mounted with glycerol buffered mounting media or DAPI. Images were captured using an Olympus laser scanning confocal microscope equipped with epifluorescence (100x magnification; resolution at 512x512 and scanning step size 1µm) and a Zeiss Axioskope fluorescent microscope. DAPI- labeled nuclei were counted (400X) along the epidermis within an arbitrarily fixed grid area (100 micron^2^) and averaged in three-sections/sample (n=4).

For samples harvested at day 7 following wounding, we divided our analysis into three separate areas: the wound itself (that had the maximum thickness of epidermal cells; greater than 0.10 mm) healing by secondary intent, the adjacent wound margin (medium thickness 0.035-0.090 mm) and nearby normal skin (<0.035 mm). Epidermal fibers (individual single axon profiles; the axon profiles form endings within the epidermis) were counted in five adjacent fields of six sections for a total 30 fields per mouse for nearby normal skin, three adjacent fields of 5 sections for a total of 15 fields for the adjacent margin medium thick zone and for the zone of maximum thickness or prior wound center. Both vertical (trajectory approximately 90° to the surface of the skin, or greater than 45° from the horizontal) and total (all single axon profiles at any orientation or angle) were analyzed by a blinded examiner, to calculate axon profile densities. The percentage of hair follicles with or without PGP9.5-labeled axons were counted in horizontal planes of each section (n=4-5 for each time point; ImageJ software, NIH, Bethesda, MD).

### (iii) qRT-PCR

Skin samples were treated with RNAseZap (Applied Biosystems Canada), flash frozen and extracted immediately using Trizol (Invitrogen). qRT-PCR used matched amounts of total RNA to synthesize first-strand DNA using SuperScript II First-strand Synthesis kit (Invitrogen) as in previous work [15,19]. Random hexamers and first-strand DNA were used and the amplified product quantitated by fluorescence of SybrGreen (Invitrogen) binding. The cycle number was determined at a fixed threshold (threshold cycle [CT]). The primer sequences used were designed in Primer Express 2.0 (Applied Biosystems, Foster City, CA) and are given:

INOS m F 5’-GCCACCAACAATGGCAACA-3’

INOS m R 5’-GTACCGGATGAGCTGTGAATT-3’

Lgr6 mF5’-CTCAACCCTTCGATCCTTGGTT-3'

Lgr6 m R5'-TTCCCTTTGAGCTTCAGGTGC-3'

We used the Ct value as the cycle number at which the fluorescence passes the threshold and calculated 100/Ct to provide a relative value comparable between groups. Between diabetic and nondiabetic mice, we confirmed a lack of change with two separate housekeeping genes (RPLP and Serpin) in 7 day wound samples.

### (iv) Western blot

Thirty µg of total protein was loaded for SDS-PAGE. The proteins were transferred on PVDF membrane in Tris-glycine-methanol buffer at 4°C for 1h. After blocking in 5% nonfat dry milk (in Tris-buffered saline containing 0.5% Tween-20 (TBST)), the membrane was incubated overnight with the respective antibodies: 1.PTEN;1:1000 in 2% BSA in TBST(2% TBST); mouse monoclonal; Cell Signaling, Danvers, MA; 2.RHOA; 1:500 in 2% TBST; mouse monoclonal; Cytoskeleton; 3.Rac1;1:400 in 2% TBST; rabbit polyclonal; Santa Cruz. Actin (1:2000 in 2% TBST; mouse monoclonal; Millipore) was used as the loading control. Horseradish peroxidase (HRP) -labeled secondary antibodies (anti-rabbit and anti-mouse IgG HRP (Santa Cruz)) at 1:2000 dilution were used. Signal detection was performed using enhanced chemiluminescent reagent (PerkinElmer, USA) exposed on X ray film (Thermo scientific, USA). Quantification of bands was done using Adobe Photoshop and the band densities were normalized with that of the loading control.

### (v) Analysis

Results are presented as means±sem. Statistical analysis used Student’s t-tests to compare diabetic and nondiabetic groups. For multiple groups in the analysis of western immunoblots we used one-way ANOVA with Tukey posthoc analysis.

## Results

### (i) Diabetic model

**Figure 1:**
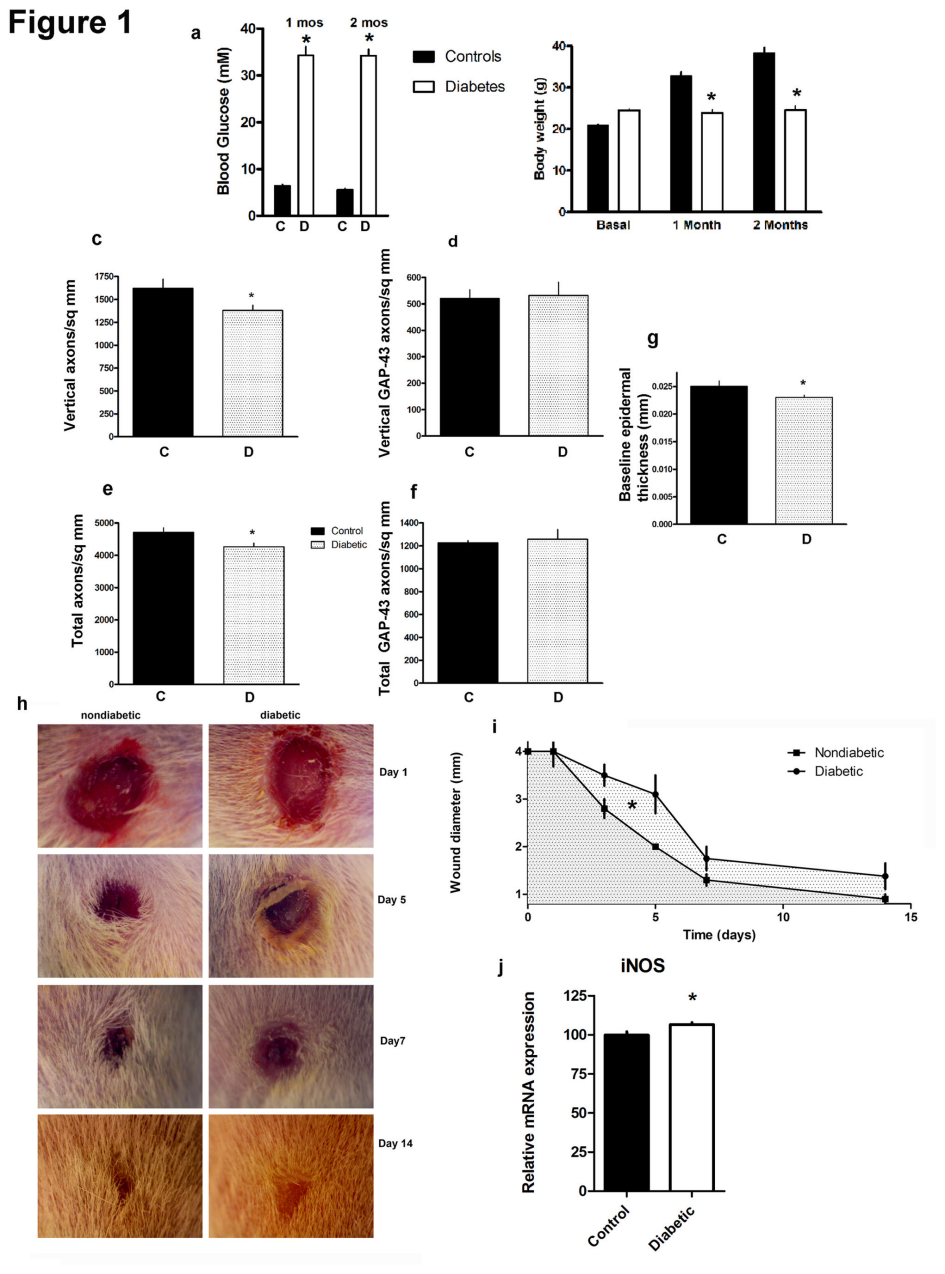
Diabetic mice (n=16) developed hyperglycemia (a) and gained less weight than nondiabetic mice (n=10) (b) [*p<0.0001]. Diabetic mice had fewer PGP 9.5 labelled epidermal axon profiles (c,e)[*p<0.05 for total axons; for vertical axons diabetic vs. nondiabetic p=0.04, one tailed t-test; n=5 diabetic, 5 nondiabetic] but no difference in GAP43 profiles (d,f) (n=3/group). Diabetics had a mild reduction in baseline epidermal thickness [p=0.05 one-tailed Student’s t-test; n=5/group] (g). At days 3-7 there was a slower decline of wound diameter in diabetics [*p<0.05 diabetic (35.6±3.7) vs. nondiabetic (26.6±1.1) areas under the curve and also separately for Day 5 diameter (n=5 diabetic, 5 nondiabetic )] (h,i). At 7d, there was a small rise in the mRNA expression of iNOS in diabetic mice[*p<0.05; n=4/group] (j). Note C=control nondiabetic; D=diabetic.

Diabetic mice developed hyperglycemia [Figure 1a] and gained less weight than nondiabetic controls [Figure 1b] (n=16 diabetic, n=10 nondiabetic). In separate work, we have shown that this model and background mouse strain is associated with electrophysiological and behavioral features of neuropathy [18]. Axon profiles in the epidermis can have different orientations. We quantified the total of all orientations and a subset that are only oriented vertically with respect to the epidermal surface. After 2 months of diabetes, there was an approximate 10-15% decline in total (axon profiles travelling in any trajectory angle) and vertically directed (arbitrary direction of at least 45° from the horizontal) density of epidermal axon profiles in the dorsal hairy skin [Figure 1c,e]. This degree of axon profile loss was as expected from previous work and less than seen in distal extremity skin such as the toe pad. There were no differences in the density of GAP43 axons, which constituted approximately one third of the overall axon population in the epidermis [Figure 1d,f] (n=3/group). The thickness of the epidermis was slightly reduced in diabetic mice at baseline [Figure 1g] (n=5/group).

### (ii) Diabetes impairs secondary intention healing

**Figure 2:**
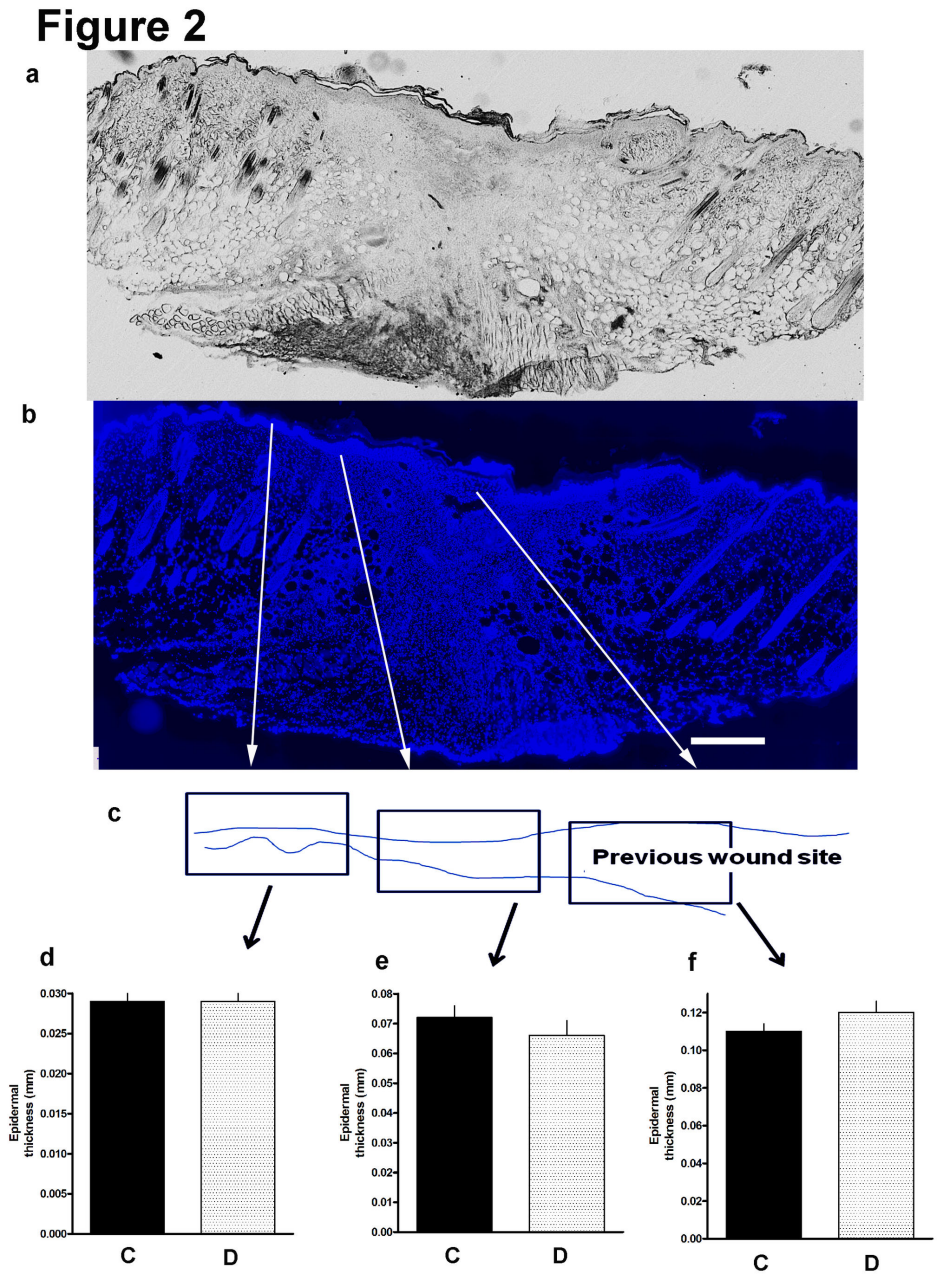
Whole mount sections through the area of wounded skin and examined under light microscopy (a) or, of the same section with DAPI staining of nuclei (b) include a central disorganized zone, the wound margin and nearby adjacent normal epidermis (white arrows) [Bar=500 microns]. The schematic shows the variation of epidermal thickness from normal skin [left arrows; left and right sides of the Figure] to the new tissue ingrowth zone [right arrow; center of the Figure] (c) is matched to tissue zones with normal appearing epidermis and dermis (d), epidermis and dermis directly adjacent to the wound site (e) and new tissue growing by secondary intent into the previously biopsied zone (f). There were no differences in thickness of these zones at 7d following injury between nondiabetics and diabetics (solid bar=nondiabetics, hatched=diabetics; n=5 diabetic, 5 nondiabetic). Note C=control nondiabetic; D=diabetic.

We explored whether cutaneous wound healing involving a type 1 model replicated the delay in healing expected of diabetes. After a 3 mm punch biopsy, we serially measured the wound size. Diabetes was associated with a slower decline in wound size most prominent at the 5-7 day mark [Figure 1h,i] (n=5/group) and a relative rise in iNOS mRNA at day 7 in the adjacent wound margin [Figure 1j] (n=4/group). At 7 days following skin wounding, we divided dorsal cutaneous wounds into three histological zones for analysis: (i) normal nearby tissue beside the wound; (ii) a marginal zone adjacent to the wound with a distorted but intact epidermal and dermal structure; (iii) a central healing core of new tissue that did not have recapitulation of epidermal and dermal architecture. These areas of analysis were associated with epidermal cell invasion that progressively thickened closer to the wound site and lost its normal architecture in a thick zone representing the previous wound site. Diabetic and nondiabetic wounds had similar epidermal thickness in all three zones [Figure 2a-f] (n=5/group). By 14 days, wounds had nearly closed in both diabetic and nondiabetic mice. To evaluate innervation, we then chose the 7d endpoint to correlate axon ingrowth with the time range of impaired healing.

### (iii) Diabetes is associated with retraction of wound margin innervation

**Figure 3:**
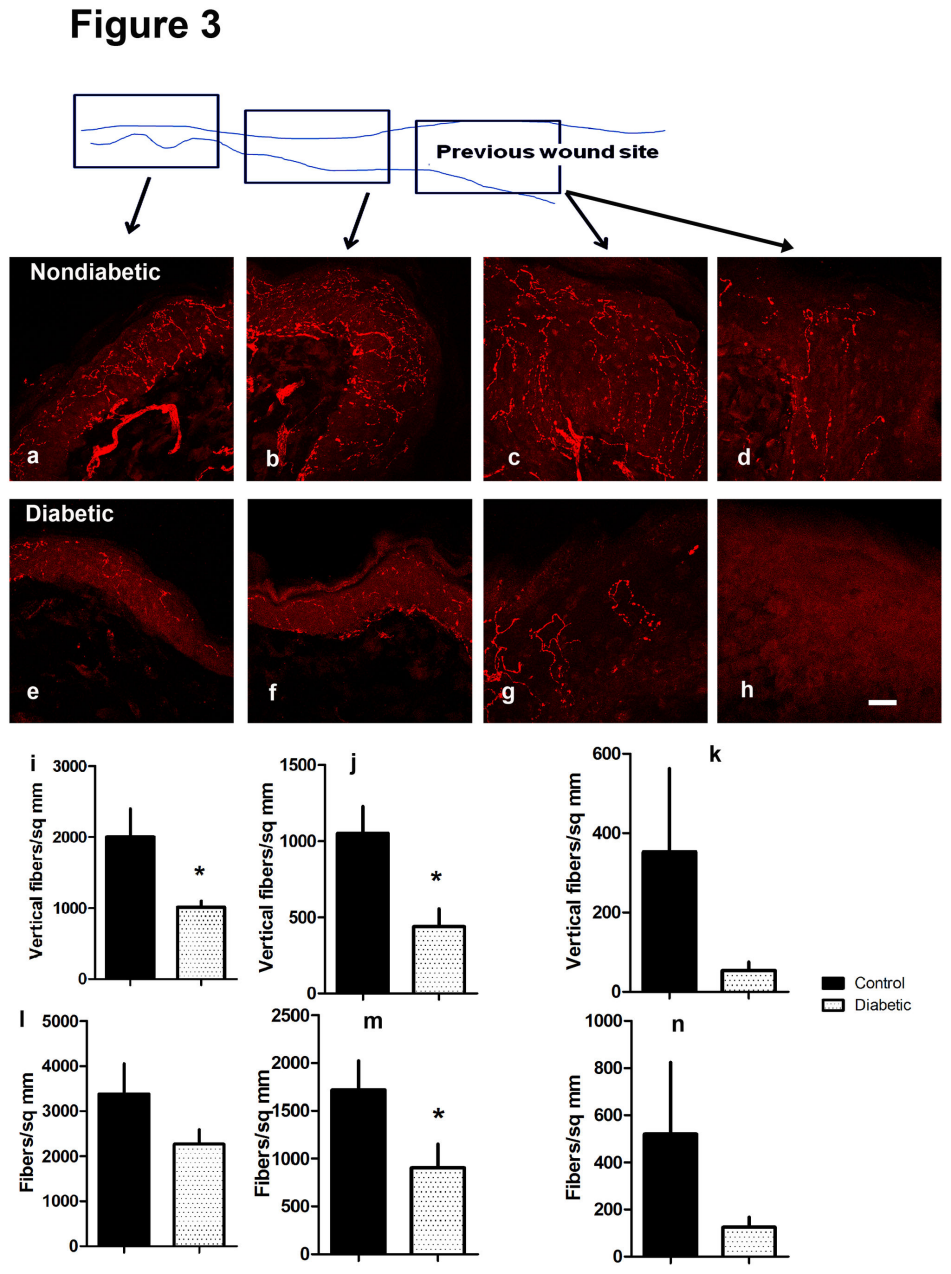
Innervation of three zones associated with dorsal skin wounds (black arrows) shown by the schematic to be from nearby normal skin (a,e), adjacent wound margins (b,f) and the wound zone healing by secondary intention (c,d,g,h). Images are labeled with an antibody directed toward PGP 9.5, an axon marker. Images (a-d) are nondiabetic littermate controls and (e-h) from diabetic mice. Data are matched to the wound zone in the micrographs above them (i,l nearby normal skin; j,m adjacent wound margin; k,n wound zone). Graphs show vertically directed axon profile density (i-k) and total axon profile density (l-n) in diabetics (open bars) and nondiabetics (black bars) [i,j,m *p<0.05 diabetic vs. nondiabetic; *p<0.05 one tailed Student’s t-test diabetic vs. control; n=4 diabetic, 4 nondiabetic]. Bar=20 microns.

Since the skin wounds healed by secondary intention, we explored both how axons accompany ingrowth of new tissue in the central core of the injury and how axons fared in the adjacent margins of the wound. Axon counts progressively declined from the nearby normal skin to within the wound margins and in the central area of the wound at the 7d time point [Figure 3].

We examined intact nearby skin, adjacent wound margins and wound secondary tissue ingrowth for their degree of innervation by 7d, a timepoint just beyond that documented for maximally impaired skin closure. To estimate the full complement of axons and their branches within the epidermis, we analyzed, as in previous work, both the density of PGP 9.5 vertically directed axon profiles (at forty-five to ninety degrees to the horizontal skin surface) and all PGP 9.5 epidermal axon profiles. Diabetes was associated with a substantial decline in vertically oriented axon profile innervation within nearby normal adjacent skin and wound margins and a decline in total axon profile density in the wound itself (n=4/group) [Figure 3]. The deficit in axon innervation had a more rapid decline from adjacent skin to core wound tissue in diabetic mice.

### (iv) Diabetes impairs local axon plasticity

**Figure 4:**
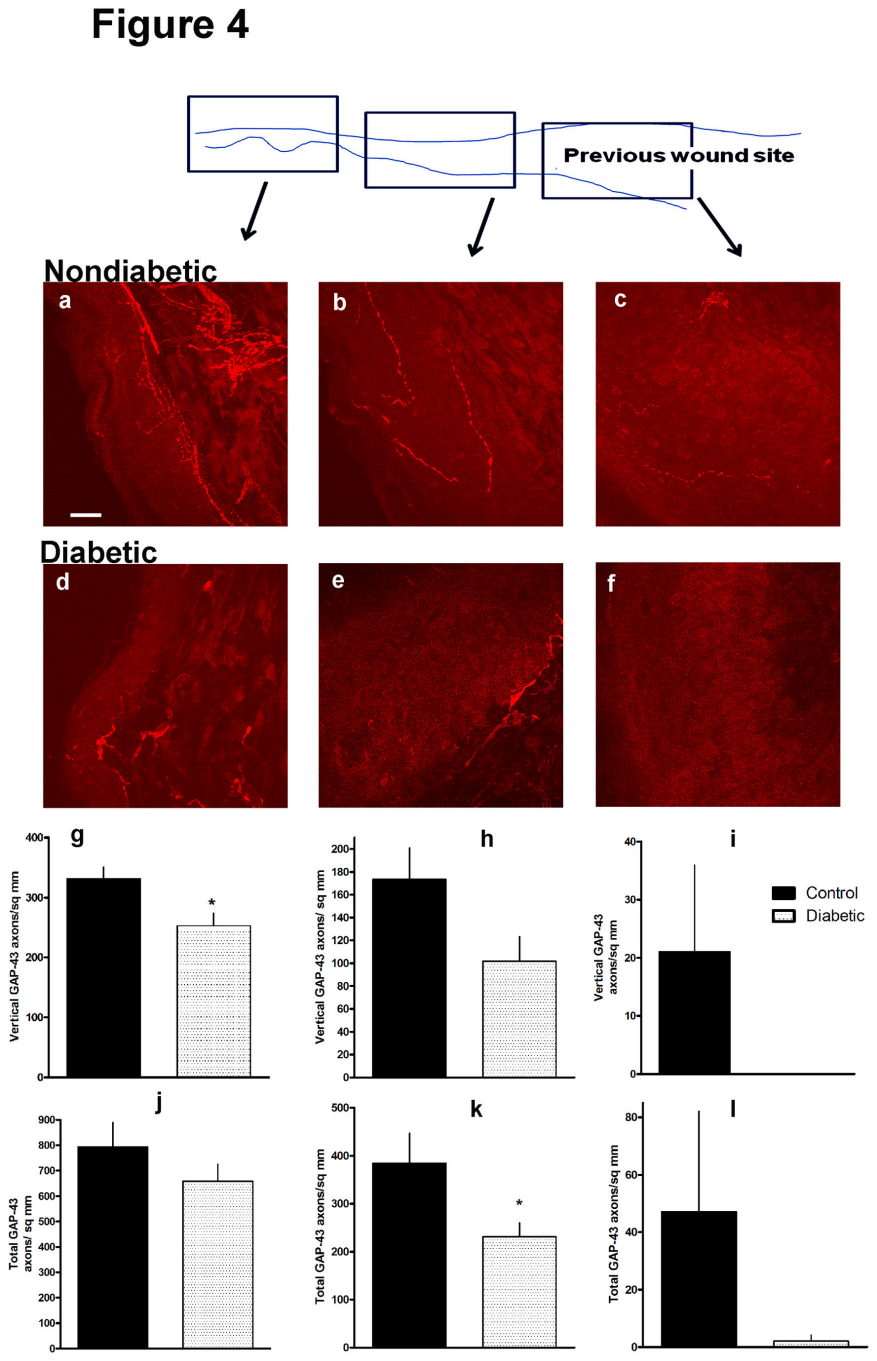
GAP43 axon innervation of three zones associated with dorsal skin wounds (black arrows) shown by the schematic to be from nearby normal skin (a,d), adjacent wound margins (b,e) and the wound zone healing by secondary intention (c,f). Images (a-c) are nondiabetic littermate controls and (d-f) from diabetic mice. Data are matched to the wound zone in the micrographs above them (g,j nearby normal skin; h,k adjacent wound margin; i,l wound zone). Graphs show vertically directed GAP 43 axon density (g-i) and total GAP43 axon density (j-l) in diabetics (open bars) and nondiabetics (black bars). [g *p<0.05; h p=0.06 (NS) diabetic vs. nondiabertic; k *p<0.05 diabetic vs. nondiabetic, one tailed Student’s t-test; n=3 diabetic, 3 nondiabetic]. Bar=20 microns.

We analyzed axon plasticity by counting axon profiles labeled with the protein GAP43/B50, a growth-related marker of axon plasticity, previously linked to epidermal innervation [22]. In parallel to the loss of axons labeled by PGP 9.5, there were significantly fewer vertically directed and total GAP43 axon profiles in diabetic mice within the nearby normal skin, and total axon profiles in the margin of the wound. These counts fell to almost zero in diabetics within the secondary ingrowth zone, compared to a small but definite influx of axon profiles in control mice [Figure 4a-l]. Taken together the findings indicated that in diabetes, axons expressed less plasticity than in nondiabetics.

### (v) Loss of perifollicular innervation and associated stem cells

**Figure 5:**
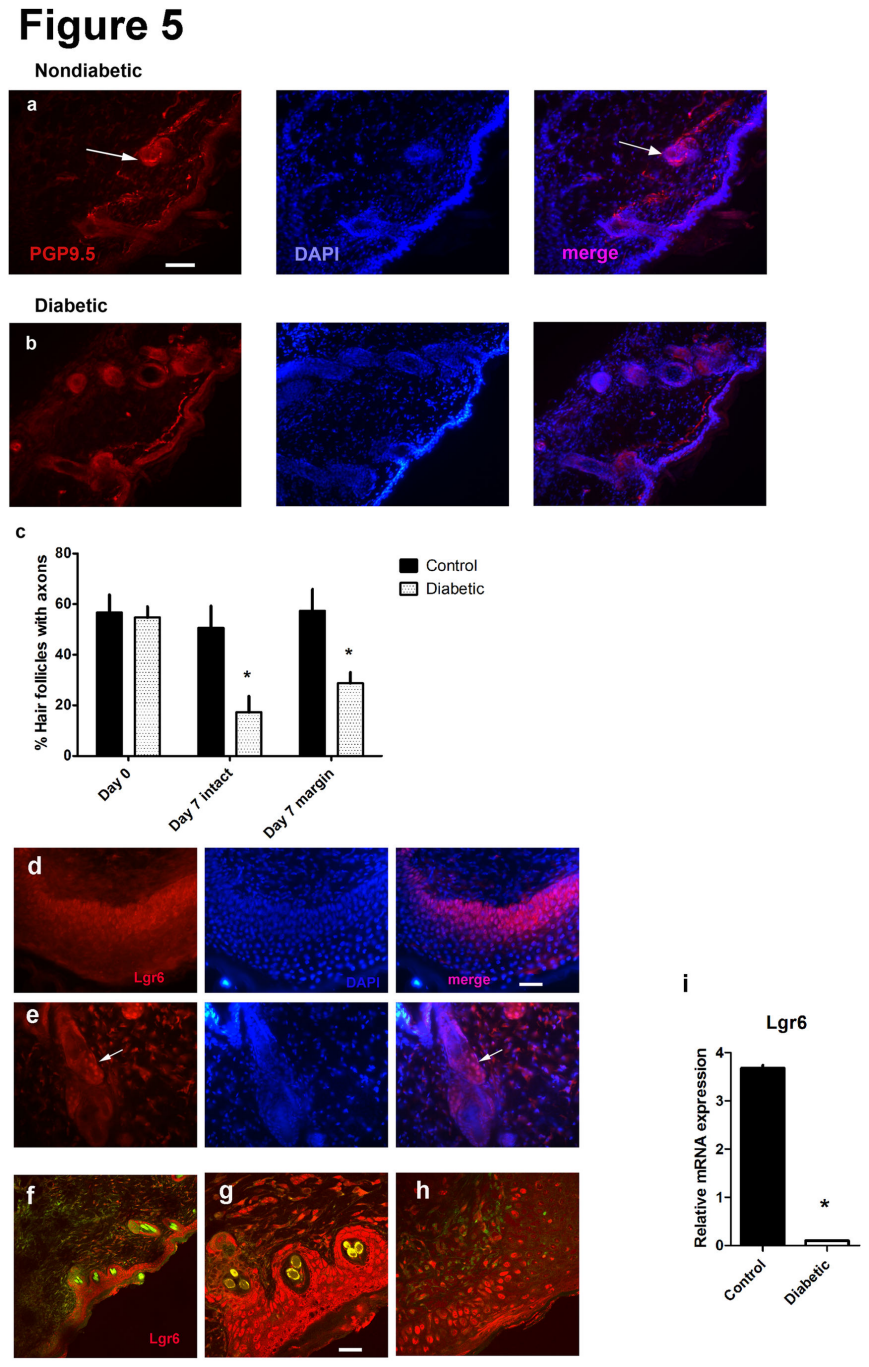
PGP 9.5 axons at day 7 are directed around the intradermal hair shaft (arrow) in nondiabetics (a) with only one subepidermal axon but no perifollicular axons in diabetics (b). [Bar=100 microns]. Quantitation indicates fewer diabetic follicular axons [*p<0.05; n=5 diabetic, 5 nondiabetic at baseline; n=4 diabetic, 4 nondiabetic at day 7] (c). The appearance of Lgr6 protein expression was similar in epidermal keratinocytes and surrounding hair follicles roots (nondiabetic mice d,f,g, at day 7 and e at baseline;Lgr6 red, DAPI nuclei blue; yellow hair shaft autofluorescence f,g). Lgr6 keratinocytes migrated into secondarily healing wound zones (h, diabetic) [Bar= 50 microns for a-f, 100 microns for g, 20 microns for h]. In diabetes at day 7 there was a reduction in Lgr6 mRNA (i) [*p<0.0001; n=4 diabetics, 6 nondiabetics].

We have previously used an approach to measure the axonal innervation of hair follicles [21]. Innervation of pelage hair follicles is found circumferentially at the follicular network A (FNA), at the dermal epidermal junction and around the isthmus of the hair follicle below the sebaceous gland (FNB) [23,24]. In this work, we examined the percentage of hair follicle profiles in which the plane of section, largely transverse, included an axon plexus, emphasizing FNB. The overall density of hair follicles was larger in diabetic mice than nondiabetic controls (data not shown), likely reflecting the smaller size of the diabetic mice. Despite this finding however, the percentage of innervated hair follicles at baseline was identical (n=5/group). However, at 7d following biopsy, the hair follicles in the wound margins and nearby normal skin of diabetic mice had an approximately 70% decline in the percentage of axons associated with them [Figure 5a-c] (n=4/group). In addition, we analyzed skin samples from the wound at 7d for their expression of Lgr6, a marker of skin stem cells. These cells are noted to be upregulated during skin regeneration and provide specific identification of a population important for epidermal regrowth [25]. We identified populations of Lgr6 epidermal cells in the basal layers of skin adjacent to wounds [Figure 5d], around hair follicles [Figure 5e-g] and infiltrating into secondarily healing wounds [Figure 5h] both in diabetic and nondiabetic mice. Levels of Lgr6 mRNA were substantially lower in diabetic skin wound samples taken from the adjacent wound margin [Figure 5i] (n=4 diabetics, 6 nondiabetics).

### (vi) Diabetes and diabetic wounds upregulate PTEN and RHOA

**Figure 6:**
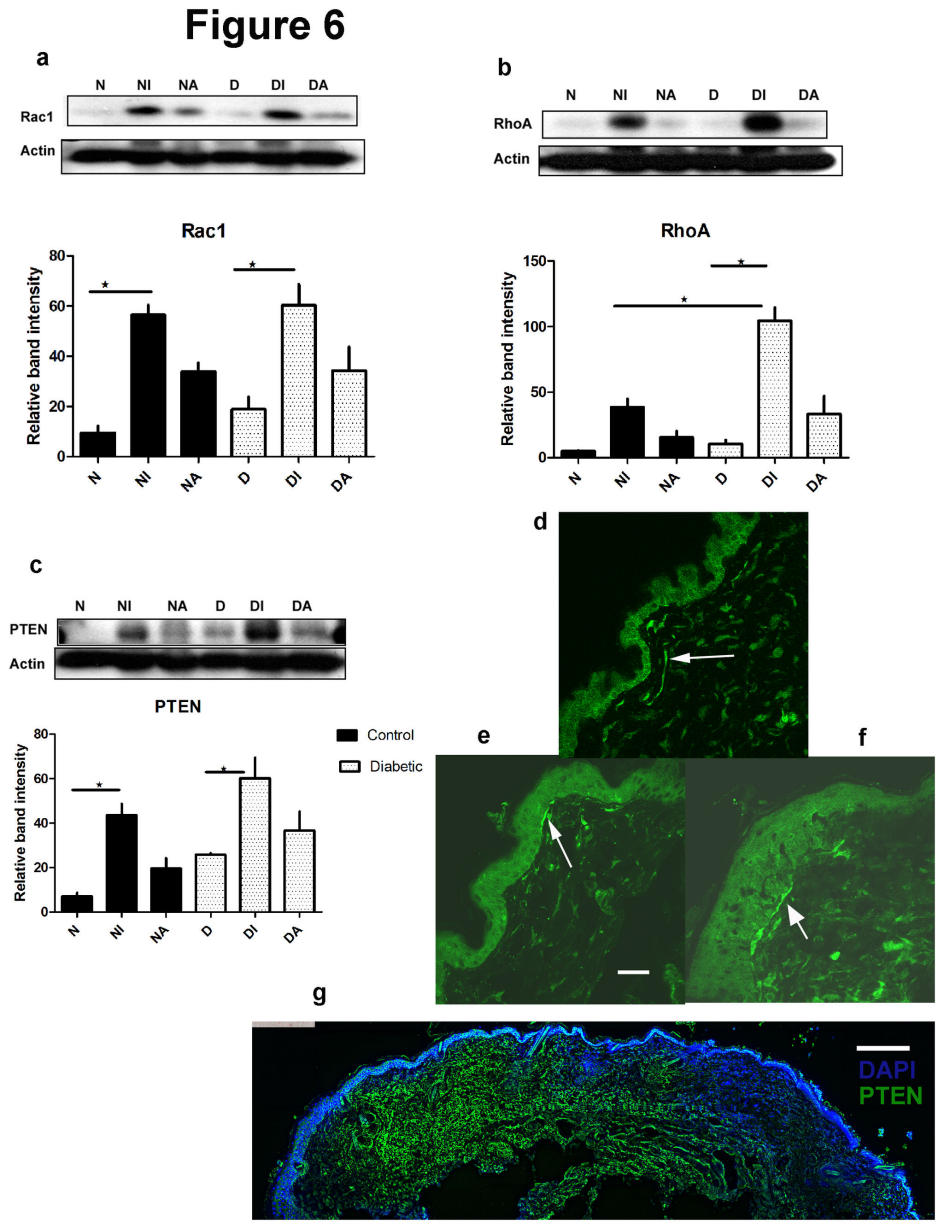
Rac1 (a) was elevated in day 7 wounds (N - nondiabetic,D-diabetic; NI,DI-injury wound zone; NA,DA- adjacent to wound) [ANOVA p=0.0004; post hoc *p<0.05 for N vs. NI and D vs. DI; n=3/group]. RHOA (b) increased in diabetics following injury [ANOVA p<0.0001; post hoc *p<0.05 for NI vs. DI, D vs. DI; n=3/group]. PTEN (c) was elevated after injury [ANOVA p=0.0006; post hoc *p<0.05 for N vs.NI and D vs. DI; n=3/group]. PTEN was localized in subepidermal axons (arrows) and diffusely in keratinocytes and dermal connective tissue (nondiabetic intact skin (d) or adjacent to wound (e) and diabetic adjacent to wound (f)) [Bar=33 microns]. There was widespread PTEN rise in the central wound zone and adjacent skin [nondiabetic illustrated by wholemount (g) [Bar=200 microns].

In previous work, we have identified three specific molecular determinants of peripheral sensory axon plasticity: Rac1, a GTPase that facilitates growth [21] and RHOA and PTEN, both intrinsic ‘brakes’ to regrowth [14,15]. Specifically, we have linked increases in epidermal axon growth with local rises in Rac1 protein levels. In this work we found that Rac1 increased by similar levels in the injury area and further adjacent skin in both nondiabetics and diabetics [Figure 6a] (n=3/group). In the case of RHOA, there were rises in both diabetic and nondiabetic skin wound zones [Figure 6b] (n=3/group). In diabetes, there was a nonsignificant trend toward elevated baseline levels of RHOA. After injury however, rises in RHOA levels in diabetes were approximately double that of nondiabetics at the injury zone. In the case of PTEN at baseline there was a nonsignificant trend toward elevated levels in diabetes. There were 3-4 fold rises in expression at the injury site compared to intact skin in both nondiabetics and diabetics [Figure 6c] (n=3/group). Adjacent skin had nonsignificant trends toward higher PTEN levels. PTEN expression was identified in several dermal connective tissue cells types including dermal axons [Figure 6d-g].

## Discussion

The major findings from this work were: (i) dorsal skin biopsy punch wounds in mice with 2 months of diabetes duration healed more slowly than similar wounds in nondiabetic littermates; (ii) the margins of wounds in diabetic mice underwent an enhanced loss of epidermal axons both immediately adjacent to the wound and within nearby normal skin; (iii) in addition to the loss of pre-existing axons, diabetic mice exhibited a failure of plasticity to send axons into newly reconstituted wound tissue; (iv) perifollicular axons were lost in skin both adjacent to the area of the wound and in adjacent nearby normal skin of diabetic mice; (v) loss of axons was associated with a decline in the expression of the mRNA for the stem cell marker Lgr6 mRNA; (vi) cutaneous wounds, but especially those in diabetics had substantial rises in their local content of PTEN and RHOA, two major roadblocks of axon and tissue plasticity.

In 12 type 2 diabetic and 12 control subjects, Krishnan et al [26] described declines in prewounding dermal skin fiber densities, measures of VEGF and of maximum flare, or hyperemia in diabetes. Biopsies from human diabetic skin ulcers with or without clinical sensory neuropathy by Galkowska et al [27] had a lack of epidermal axons, severe loss of dermal axons, loss of CGRP axons in neuropathic patients and a low grade inflammatory reaction. Gibran et al noted fewer epidermal and papillary dermal axons from human diabetic subjects [28]. Nondiabetics were not compared in these latter two studies. Current consensus approaches toward the quantitation of the density of axons in human skin biopsies are also now more detailed [29].

Our own counts, involving the mouse model, were more extensive and captured branched profiles by examining both vertically directed and total axon profile densities. In our diabetic model, axon profile densities were 10-15% reduced in the epidermis at baseline, but up to 50% fewer around wounds at day 7. No such loss was identified around nondiabetic wounds. It is remarkable that this degree of axon loss accompanied a relatively mild delay in healing in this model, resolved by 14 days. In more extensive wound models, we suspect that this finding may be yet more extensive.

The impairments of diabetic skin wound healing have many parallels in how diabetic peripheral nerve trunks respond to injury. Injured diabetic nerve trunks have a delay in their influx of macrophages [30], an attenuation of injury-related hyperemia, and impaired angiogenesis in response to injury [31]. Finally, regeneration of nerves through a nerve gap following transection, is severely disrupted by diabetes [32]. In parallel work, reforming nerve trunks across transection gaps were severely disrupted if forced to regrow through areas of intense but sterile inflammation [33] linked in part to local elaboration of NO generated by iNOS. Classical assumptions might link axon retraction to wound edge ischemia and microvascular impairment. However, this explanation is unsatisfying, requiring that a critical level of ischemia would selectively account for extensive withdrawal of axon terminals while adjacent tissue was repaired, although a little more slowly. Toxic damage from excessive elaboration of oxidative or nitrosative radicals is a possibility. In support of this, we did observe rises in iNOS mRNA, a robust marker of tissue inflammation from macrophages and other inflammatory cells, that were more striking in the diabetic wounds. How exactly this targets nerve terminals selectively is uncertain.

What is clear from the present and previous work is that cutaneous innervation is not static but constantly remodeling and subject to several plasticity cues. The short term rises in innervation both within epidermis and around hair follicles secondary to noninvasive shaving supports this concept [21]. Similarly, low doses of insulin applied subdermally reversed loss of footpad epidermal terminals in type 1 and 2 diabetic mice [17]. The perifollicular fiber loss is particularly important as it relates to how follicles, and their stem cell populations, like those labeled with Lgr6, support the regrowth of skin. We believe that there are hitherto undefined relationships between these local cell populations and the axons associated with them.

Overall, a normally dynamic innervation may be capable of retracting or advancing its terminals in response to local molecular signals. Our choices of proteins to analyze were based on their demonstrated roles in facilitating or inhibiting axon growth. The expression of GAP43 in cutaneous axons, observed in a large proportion of these fibers normally, is an indication of that plasticity. In diabetes, GAP43 axons were dramatically reduced in numbers both around the wound sites and into new tissue as new nerve sprouts. Rac1 acts downstream of HGF and enhances growth cone advance [21]. In the case of PTEN, an intrinsic ‘brake’ to PI3K-pAkt signaling is erected, attenuating an important and well established regenerative signal. PTEN expression was prominent in axons but expressed in other cell types, where it may impose similar barriers to cell migration and plasticity during normal skin remodeling or after wound repair. For example, PTEN inhibition enhances corneal wound healing [34]. Similar barriers may be erected by RHOA [15], also upregulated in the wounds we analyzed and known to interact with PTEN [35]. At this stage, the observed reductions in axon counts however are strictly associations and despite the trends, rises for PTEN were not statistically significant between diabetics and controls. Critical functional analysis, not within the scope of the present work, is required to further these observations. For example it would be of significant interest to understand whether pharmacologically inhibited or genetically deleted PTEN or RHOA would repair deficits in wound healing in diabetic mice whilst improving the innervation of healing and adjacent skin.

There are other important caveats in this work. Diabetic ulcers are often found in the lower limbs where vascular compromise and pre-exisiting neuropathy are likely to be more severe than in the dorsal skin we examined [7]. Similarly, diabetic foot ulcers are also complicated by superimposed infection and they are chronic, rather than acute lesions [36] [26]. Overall, the concept that there is active retraction of pre-existing axons from the wound and its environs, rather than a mild pre-existing deficit, has important implications for how axons fare in diabetes and how they might contribute toward wound repair. While these findings are associative at this time, they support the concept that sensory innervation and local peptide release may have key links to altered healing. What is remarkable is that only minor deficits in dorsal skin innervation in diabetic mice develops into substantial loss after wounding. How this dramatic change in axon plasticity occurs requires further work.
